# Pyriform sinus perforation during nasotracheal intubation: a case report

**DOI:** 10.1016/j.ijscr.2020.06.032

**Published:** 2020-06-13

**Authors:** Mohammed Elrabie Ahmed, Bahaa Mohammed Refaie, Farghali Abdelrahman, Abdul-Rahman Abdul-Majeed Ragab

**Affiliations:** a Otolaryngology - Head and neck surgery Department, Faculty of Medicine, Sohag University, Egypt; b Anesthesia and ICU Department, Faculty of Medicine, Sohag University, Egypt; c Radiology Department, Faculty of Medicine, Sohag University, Egypt

**Keywords:** Pyriform, Perforation, Intubation, Complication

## Abstract

**Introduction:**

Endotracheal intubation is the gold standard to protect the airway during general anesthesia. It is the commonest technique in medical practice with a low complication rate.

**Presentation of case:**

We report a case of left pyriform fossa perforation in a child after apparently uneventful nasotracheal intubation for tonsillectomy.

**Discussion:**

This is a rare complication. Physicians performing endotracheal intubation or dealing with intubated patients should be aware of the clinical symptoms of such complications. A rational management approach has been discussed.

**Conclusions:**

Early diagnosis is the mainstay to improve the outcome and avoid the morbidity and potentially life-threatening course of such trauma.

## Highlights

• Endotracheal intubation is the commonest technique in medical practice with a low complication rate.
• Physicians performing endotracheal intubation or dealing with patients after intubation, should be aware of the clinical symptoms of such complications.
• Early diagnosis is the mainstay improving the outcome and avoids the morbidities and life-threatening course potentially.

## 1 Introduction

Endotracheal intubation is a very common worldwide procedure in anesthesia and emergency. It is considered a relatively safe procedure. Currently, even paramedical staff encouraged for endotracheal intubation. Despite training and thorough preparation failed or traumatizing intubation may be associated with a rare major complication in the form of hypopharyngeal or esophageal perforation [1]. Iatrogenic injury of the upper aerodigestive tract is infrequent but a serious complication, which may lead to substantial morbidity and mortality. Anesthetists must be aware of this potential injury, especially when difficult airway is encountered, associated airway trauma, and/or persistent patient complaints. Perforations in the hypopharyngo-esophagus may result in mediastinitis, even with modern antibiotics, it is still a life-threatening complication [2]. We present a case with left pyriform sinus perforation following uneventful nasotracheal intubation. We tried to focus on the proper diagnosis and management of such cases. This case report has been reported in accordance to the Surgical Case Report (SCARE) guidelines [3].

### 1.1 Presentation of case

**Fig. 1:**
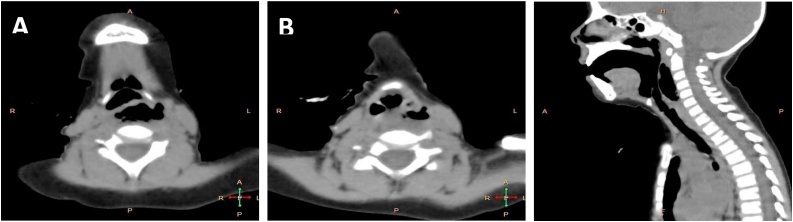
Neck CT; Axial (A,B) and Coronal (C) revealed air retropharyngeal and left parapharyngeal.

**Fig. 2:**
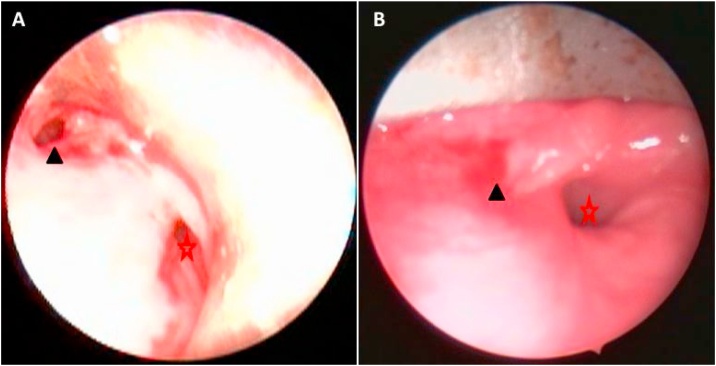
Endoscopic view of the hypopharynx; (A) showing perforation site at left pyriform (black Arrow head); (B) healing of left pyriform perforation. Red star showing the upper esophageal opening.

A three years old male child presented with odynophagia with drooling of saliva immediately after tonsillectomy. According to the anesthesia report, nasotracheal intubation using direct laryngoscopy was atraumatic and uneventful but airway assessment for difficult intubation using the Mallampati or Cormack and Lehane scores was not specified in the report. The child admitted for two weeks in another hospital before transfer to our tertiary hospital after the failure of medical treatment. Neck examination showed left side small collection and localized left side emphysema. Full Laboratory investigations were done (HB 8.6 mg/dl; WBC 7.8 × 1000 cm^3^; ESR 1st hour 51 min 2nd hour 84 min; CRP 4.7). A blood transfusion was given. Neck lateral radiograph and Computer tomography of the neck and chest with contrast were done showing retropharyngeal emphysema with no evidence for tear Fig. 1. The endoscopic evaluation revealed left pyriform perforation with purulent discharge, so suction was done, and a nasogastric tube was used for feeding Fig. 2. Follow up neck lateral radiograph showed no improvement of retropharyngeal collection, so the child underwent external drainage with a drain insertion. Three weeks later, Gastrografin swallow could not be done as the child refuse to swallow the dye. Endoscopic evaluation was done again and revealed a small opening in the left pyriform and Betadine injection through it revealed a pharyngocutaneous fistula. Refreshing of the edges with chemical cauterization and fibrin glue injection were done. The patient was symptom-free and released home in good general condition. The patient admitted for 4 weeks before discharge. A follow-up examination after 3 months yielded no pathological findings.

### 1.2 Discussion

Endotracheal intubation is usually atraumatic whether it was performed as an emergency or elective procedure [4,5]. However, on rare occasions intubation perforations injuries with high morbidity and mortality may occur [[6], [7], [8]]. As the intubator usually plays only a transient role in the patient’s care, iatrogenic pharyngoesophageal perforations may go unsuspected until characteristic signs and symptoms become evident [9].

Nasotracheal intubation is often requested by surgeons for patients undergoing otolaryngologic or maxillofacial surgery as an orotracheal tube may interfere with surgical exposure and associated with dislodgement risk during surgery. Yet nasotracheal intubation is not without risks. Epistaxis is the most common complication ranging from 10–80%, dislodgement of the adenoid or concha, sinusitis, and bacteremia. Less commonly, pharyngoesophageal mucosal dissection or perforation and inadvertent intracranial placement have been documented [10].

The most vulnerable parts to the intubation injury are the posterior pharyngeal wall, the piriform sinus, and the cervical esophagus [8].

Intubation by inexperienced personnel especially in an emergency as during resuscitation efforts under poor conditions or in cases with unexpected difficult airway is generally agreed as the single most important risk factor for such trauma [2]. Physical exacerbating factors include the use of rigid, beveled endotracheal tubes with stylets; anatomic constraints; and improper positioning of the neck [11]. In addition, poor muscle relaxation, cricoid pressure, poor laryngeal visualization, and repeated “blind” forceful attempts all contribute to pharyngoesophageal trauma [12]. A chronic indwelling nasogastric tube and chronic steroid usage have been identified as a possible predisposing factor for perforation due to weakening and increase tissue fragility [1]. An esophageal injury can be exacerbated by inflated tube cuff, as well as by positive pressure ventilation [7].

The morbidity of this injury varies from simple cervical subcutaneous emphysema and pneumomediastinum up to pneumothorax, deep cervical abscess, mediastinitis, respiratory distress, and death. In chronic cases, hypopharyngeal pseudodiverticulum [13]. The accurate incidence of such complications is unknown but obviously low, although hypopharyngeal bruising from efforts at intubation was found in 17% of necropsies [14].

The proper management of such complications can be achieved through clinical suspicion and early recognition together with a prompt diagnosis which is done by radiological investigation and/or direct laryngoscopy [9].

The early non-specific symptoms may delay the diagnosis. The initial symptoms include neck and chest pain, cough, and dysphagia. The most common signs and symptoms that make physicians worry after intubation trauma are cervical pain, fever, leukocytosis, and dysphagia. Fever may be a late symptom and occurs as a result of a bacterial inflammation of the tissues (mostly by the mouth and throat flora) [15]. Persistent complaints of neck or thorax pain after intubation should always raise suspicion of injury to the aerodigestive tract, even after uncomplicated intubation. Other findings include subcutaneous emphysema, pneumomediastinum, pneumothorax, hemoptysis, and a cervical mass. Neural palsy of internal and external laryngeal nerve result in hypopharyngeal anesthesia with consequent aspiration and cough or a hoarse, breathy voice [14]. In our case the diagnosis was delayed (after 2 weeks) as there was no clear history of resistance or trauma during the process of endotracheal intubation, absence of intraoperative problems under anesthesia and lack of experience of such cases with the first medical team dealt with the child. The child has long hospital stay but was lucky and overcome such complications without sequels.

Once intubation injury is suspected, management should be started immediately. Oral feeding should be stopped, and the patient should receive broad-spectrum intravenous antibiotics [5]. Work-up for a pharyngoesophageal trauma should proceed immediately. Initial studies include lateral neck and chest X-rays. A chest radiograph taken immediately after the injury may be negative and doesn't exclude the possibility of perforation. So the time interval between the injury and the radiological examination should be taken into consideration [16]. The water-soluble contrast esophagogram may localize the site of injury by extravasation. When suspicion of a lesion remains high in the absence of positive evidence, computed tomography or magnetic resonance imaging may give additional information [6]. A useful investigation is a direct endoscopy which provides the accurate defect site and size. Depending on the defect size conservative or surgical management could be decided [15].

Once the diagnosis has been confirmed, considerable controversy exists concerning the best method of treatment. An editorial in the Journal of the American Medical Association in 1965 supported only antibiotic therapy and nasogastric tube [17]. Later, Groves, 1966 stated that the “proper management of iatrogenic esophageal perforation is appropriate surgery associated with postoperative antibiotic support” [18].

Berry and Ochsner, 1973 observed that 50% of the patients treated conservatively required surgical drainage one to five days after the insult [19]. Hirsch et al. have suggested better results by adopting the surgical approach, particularly after the early demonstration of the leak of water-soluble contrast medium [14].

In early diagnosed cases without local and systemic inflammatory reactions, antibiotic therapy combined with endoscopic closure of the perforation can be considered. In the postoperative period, Ryle feeding ensures healing without lasting consequences [15]. According to our local experience, the endoscopic drainage in late cases is insufficient and should be associated with surgical treatment.

Conventional repair entails a two-layer, primary suture closure of the defect with either active or passive cervical drainage. When primary suture repair of a large and/or necrotic pharyngoesophageal defect cannot be accomplished, closure using a sternocleidomastoid muscle flap has been described for reconstruction and augmentation. Nasogastric tube feeding is used for approximately 1 week or until cervical drainage ceases [14,20].

Hawkins et al. 1974 reported lethal outcome in 37.5% of upper aerodigestive perforations after endotracheal intubation [2]. Dubost et al. 1979 reported that mortality may reach 56% when surgical intervention is delayed for more than 12 h in the symptomatic patients [4]. Rapid detection and combination of surgical and antibiotic therapy are the only ways to avoid serious consequences.

Before nasotracheal intubation, anesthesiologists should examine the nasal passage, use a local decongestant, lubricate the endotracheal tubes, and try to pass the tube gently. Finally, the pharynx should be examined after nasal intubation to rule out complications [21].

## 2 Conclusion

Iatrogenic perforation of the upper aerodigestive tract is still a serious complication of endotracheal intubation. Prevention of such complications is the best achieved by the supervision and training of anesthetists and practitioners of other disciplines. Early diagnosis is the mainstay to improve the outcome and avoid the potentially life-threatening course of such trauma.

## Sources of funding

None.

## Ethical approval

This report complies with regional and institutional ethical guidelines and with declaration of Helsinki.

## Consent

A written informed consent was obtained from the parents of our case for her participation.

## Author contribution

Mohammed Elrabie Ahmed

Data collection

Study concept or design

Drafting the manuscript

Review and approval of final copy of the manuscript

Bahaa Mohammed Refaie

Writing the paper

Review of literature

Review and approval of final copy of the manuscript

Farghali Abdelrahman

Data collection

Review and approval of final copy of the manuscript

Abdul-Rahman Abdul-Majeed Ragab

Data collection

Review and approval of final copy of the manuscript

## Registration of research studies

NA.

## Guarantor

Corresponding author:

Mohammed Elrabie Ahmed

E mail: dr.mohammedelrabie@gmail.com

Mobile phone: +201,011,805,501

## Authorship

All authors attest that they meet the current ICMJE criteria for Authorship.

## Availability of data and material

Any data or materials related to the case are readily available for revision.

## Provenance and peer review

Not commissioned, externally peer-reviewed.

## Declaration of Competing Interest

The authors declare that we have no known competing financial interests or personal relationships that could have appeared to influence the work reported in this paper

## References

[1] Pacheco-Lopez P.C., Berkow L.C., Hillel A.T., Akst L.M. (2014). Complications of airway ManagementDiscussion. Respir. Care.

[2] Hawkins D.B., Seltzer D.C., Barnett T.E., Stoneman G.B. (1974). Endotracheal tube perforation of the hypopharynx. West. J. Med..

[3] Agha R.A., Borrelli M.R., Farwana R., Koshy K., Fowler A.J., Orgill D.P., Zhu H., Alsawadi A., Noureldin A., Rao A., Enam A. (2018). The SCARE 2018 statement: updating consensus Surgical CAse REport (SCARE) guidelines. Int. J. Surg..

[4] Dubost C., Kaswin D., Duranteau A., Jehanno C., Kaswin R. (1979). Esophageal perforation during attempted endotracheal intubation. J. Thorac. Cardiovasc. Surg..

[5] Levine P.A. (1980). Hypopharyngeal perforation: an untoward complication of endotracheal intubation. Arch. Otolaryngol..

[6] Ward M.P., Glazer H.S., Heiken J.P., Spector J.G. (1985). Traumatic perforation of the pyriform sinus: CT demonstration. J. Comput. Assist. Tomogr..

[7] O’Neill J.E., Giffin J.P., Cottrell J.E. (1984). Pharyngeal and esophageal perforation following endotracheal intubation. Anesthesiology.

[8] Wolff A.P., Kessler S. (1973). Iatrogenic injury to the hypopharynx and cervical esophagus: an autopsy study. Ann. Otol. Rhinol. Laryngol. [Internet].

[9] Tartell P.B., Hoover L.A., Friduss M.E., Zuckerbraun L. (1990). Pharyngoesophageal intubation injuries: three case reports. Am. J. Otolaryngol..

[10] Krebs M.J., Sakai T. (2008). Retropharyngeal dissection during nasotracheal intubation: a rare complication and its management. J. Clin. Anesth..

[11] Norman E.A., Sosis M. (1986). Iatrogenic oesophageal perforation due to tracheal or nasogastric intubation. Can. Anaesth. Soc. J..

[12] Blanc V.F., Tremblay N.A. (1974). The complications of tracheal intubation: a new classification with a review of the literature. Anesth. Analg..

[13] Hawkins D.B., House J.W. (1974). Postoperative pneumothorax secondary to hypopharyngeal perforation during anesthetic intubation. Ann. Otol. Rhinol. Laryngol. [Internet].

[14] Hirsch M., Abramowitz H.B., Shapira S., Barki Y. (1978). Hypopharyngeal injury as a result of attempted endotracheal intubation. Radiology.

[15] Luers J.C., Preuss S.F., Bovenschulte H., Mackenrodt K.A., Beutner D. (2008). Hypopharynxperforation nach endotrachealer intubation. Anaesthesist.

[16] Michel L., Grillo H.C., Malt R.A. (1982). Esophageal perforation. Ann. Thorac. Surg..

[17] Mengoli L.R., Klassen K.P. (1965). Conservative management of esophageal perforation. Arch. Surg..

[18] Groves L.K. (1966). Instrumental perforation of the esophagus: what is conservative management?. J. Thorac. Cardiovasc. Surg..

[19] Berry B.E., Ochsner J.L. (1973). Perforation of the esophagus: a 30 year review. J. Thorac. Cardiovasc. Surg. [Internet].

[20] Conley J., Gullane P.J. (1980). The sternocleidomastoid muscle flap. Head Neck Surg..

[21] Bozdogan N., Sener M., Yavuz H., Yilmazer C., Turkoz A., Arslan G. (2008). Retropharyngeal submucosal dissection due to nasotracheal intubation. Acta Otorhinolaryngol. Belg..

